# Well-Dispersed Cu_2_ZnSnS_4_ Nanocrystals Synthesized from Alcohols and Their Applications for Polymer Photovoltaics

**DOI:** 10.1186/s11671-016-1761-6

**Published:** 2016-12-13

**Authors:** Jiang Cheng, Zhongjun Dai, Bing Chen, Ran Ji, Xin Yang, Rong Hu, Jiang Zhu, Lu Li

**Affiliations:** 1 Co-Innovation Center for Micro/Nano Optoelectronic Materials and Devices, Research Institute for New Materials and Technology, Chongqing University of Arts and Sciences, Chongqing, 402160 People’s Republic of China; 2 College of Materials and Chemical Engineering, Chongqing University of Arts and Sciences, Chongqing, 402160 People’s Republic of China

**Keywords:** Nanocrystalline material, Cu_2_ZnSnS_4_, Solvothermal method, Photoelectrochemical property

## Abstract

In this work, we report on a simple non-injection synthesis routine for the preparation of well-dispersed monocrystalline Cu_2_ZnSnS_4_ (CZTS) nanoparticles (NPs). The nanocrystal morphology was investigated by scanning and transmission electron microscopy, and its phase composition was studied by X-ray diffraction and Raman analyses. Cu_2_ZnSnS_4_ nanoparticles prepared using ethanolamine and diethanolamine as chemical stabilizers showed a high purity and a suitable size for polymer solar cell applications. The fabricated CZTS NPs are shown to be easily dispersed in a polymer/fullerene aromatic solution as well as the hybrid photovoltaic active layer. Thanks to the increment in the light absorption and electrical conductivity of the active layer, solar cells with a small amount of CZTS nanoparticles resulted in a clear enhancement of the photovoltaic performance. The short-circuit current density is increased from 9.90 up to 10.67 mA/cm^2^, corresponding to an improvement in the power conversion efficiency (PCE) from 3.30 to 3.65%.

## Background

Bulk heterojunction (BHJ) polymer solar cells (PSCs) have gained interest due to their light weight, flexibility, and low-cost fabrication by using solution-processing methods [1–3]. Recently, PSCs with a high power conversion efficiency (PCE) of ~10% have been fabricated in laboratories by many groups by employing new organic photovoltaic materials including conducting polymers [4, 5] and non-fullerene (NF) acceptors [6]. Nerveless, the large-scale fabrication of commercially viable PSCs is still unsatisfactory [7, 8]. Large-scale preparation methods such as conventional doctor-blading, brush-painting, spray-coating, screen, and ink-jet printing cannot provide as efficient PSCs as laboratory-derived spin-coated photovoltaic devices. This is mainly due to the fact that these methods mentioned above are deficient for the deposition of thin organic films (100–300 nm) with the desired morphology. Typically, in a BHJ solar cell, the active layer is composed of a conjugated polymer electron donor and an organic electron acceptor compound sandwiched by interfacial layers and patterned electrodes. The active material and the interfacial contact are crucial aspects determining the photovoltaic device performance. Compared to inorganic photovoltaic materials, conducting polymers usually display sensibly lower carrier mobility. The active layer should thus be designed to be very thin in order to separate positive and negative charge carriers and collect them efficiently at the respective metal electrodes [9].

It is well-known that the charge photogeneration in PSCs can be divided into four steps: light absorption (A), exciton diffusion (ED), charge separation (CS), and charge collection (CC). The photon conversion efficiency of a PSC (*η*) can be thus described as the product of all processes efficiencies: *η*(PCE) = *η*_A_ × *η*_ED_ × *η*_CS_ × *η*_CC_[10]. The decrease of active layer thickness enhances *η*_CC_ while it lowers *η*_A_ in the visible light. This is the typical reason why PSCs usually have a lower current density (*J*_SC_) compared to inorganic solar cells. To overcome this limitation, nanostructures or nanomaterials have been employed for increasing the PCE in donor/acceptor material systems. One of the most effective ways which was reported to improve the *J*_SC_ in a thin polymer active layer is by increasing the contact interface between the active layer and the buffer layer by using ZnO-based nano-ridges or nano-flakes [11, 12]. However, Liang et al. suggested that voids can occur at the interface when ZnO has a rough surface, resulting in an increased series resistance (*R*_S_) and deceased shunt resistance (*R*_SH_) [13]. Inorganic nanocrystals (NCs) are appealing candidates for increasing both the absorption rate and the electrical conductivity of an active layer. Luszczynska et al. [14] have obtained an improvement of the maximum external quantum efficiency from 48 to 70% for a P3HT/PC_61_BM bulk heterojunction by adding Cu–In–Se NCs into the P3HT:PCBM blend. Their following work showed an improved photovoltaic performance for P3HT:PCBM solar cells by adding CuInS_2_ NCs [15]. Although the PCE was limited, the addition of metal sulfide NPs into the polymer resulted in an encouraging enhancement of the light-harvesting ability and *J*_SC_ (from 3.47 to 6.34 mA/cm^2^). Luan et al. [16] also have improved the PCE of P3HT:PCBM solar cells from 2.8 to 3.0% with adding a mount FeS_2_. The deficiency was the Low FF of the PSCs which was possibly influenced by the rough surface and poor disperse of the NPS. By optimizing the active layer morphology, there is still room for improving the PSC performance and in particular for the fill factor.

In this study, we demonstrate that the photovoltaic properties of a PSC can be improved by adding solvothermally synthesized monocrystalline Cu_2_ZnSnS_4_ (CZTS) NPs into the P3HT:PCBM active layer. The peculiarity of this study is that we have employed an advanced N_2_ protected ultrasonic spray system for improving the deposition of the hybrid organic–inorganic active layer. Firstly, we will introduce our simple and low-toxicity solvothermal method for the synthesis of single-phase monocrystalline CZTS NPs. These NPs can be well-dispersed in aromatic precursor solutions. The successful preparation of high-quality CZTS NPs results to rely mostly in choosing the appropriate chemical stabilizer and preparing the precursor solution in the right order. Thanks to the increase in the light absorption efficiency and to the higher electron mobility (~5 cm^2^ V^−1^ s^−1^) [17, 18], the PSC with CZTS NPs exhibited a considerable increase in the *J*_SC_ and the external quantum efficiency (EQE).

## Methods

### Preparation of CZTS NPs

A mixed solvent containing 40 mL ethanol, 1.5 mL diethanolamine, and 1 mL acetate was firstly prepared. Then, 0.5 mmol CuCl_2_·2H_2_O, 0.25 mmol Zn(CH3COO)_2_·2H_2_O, 0.25 mmol SnCl_2_·2H_2_O, and 1 mmol thiourea were added to the solvent under magnetic stirring. The mixture turned into a bright blue solution after the addition of 1.5 mL ethanolamine. Then, the mixture was diluted by 120 mL ethanol and transferred into 50 mL para poly phenol (PPL) lined stainless steel autoclaves with a capacity of 32 mL (80% capacity). The autoclaves were heated at 240 °C for 24 h and then naturally cooled down to room temperature. The final product was centrifuged at 13,000 rpm for 30 min and washed with ethanol repeatedly for at least three times, followed by a drying at room temperature in a vacuum oven. In order to highlight the role of the chemical stabilizer on the phase composition and morphology, three chemical stabilizers (ethanolamine, oleylamine, and diethanolamine) with specific ratios were investigated.

### Fabrication and Testing of Photovoltaic Devices

**Fig. 1:**
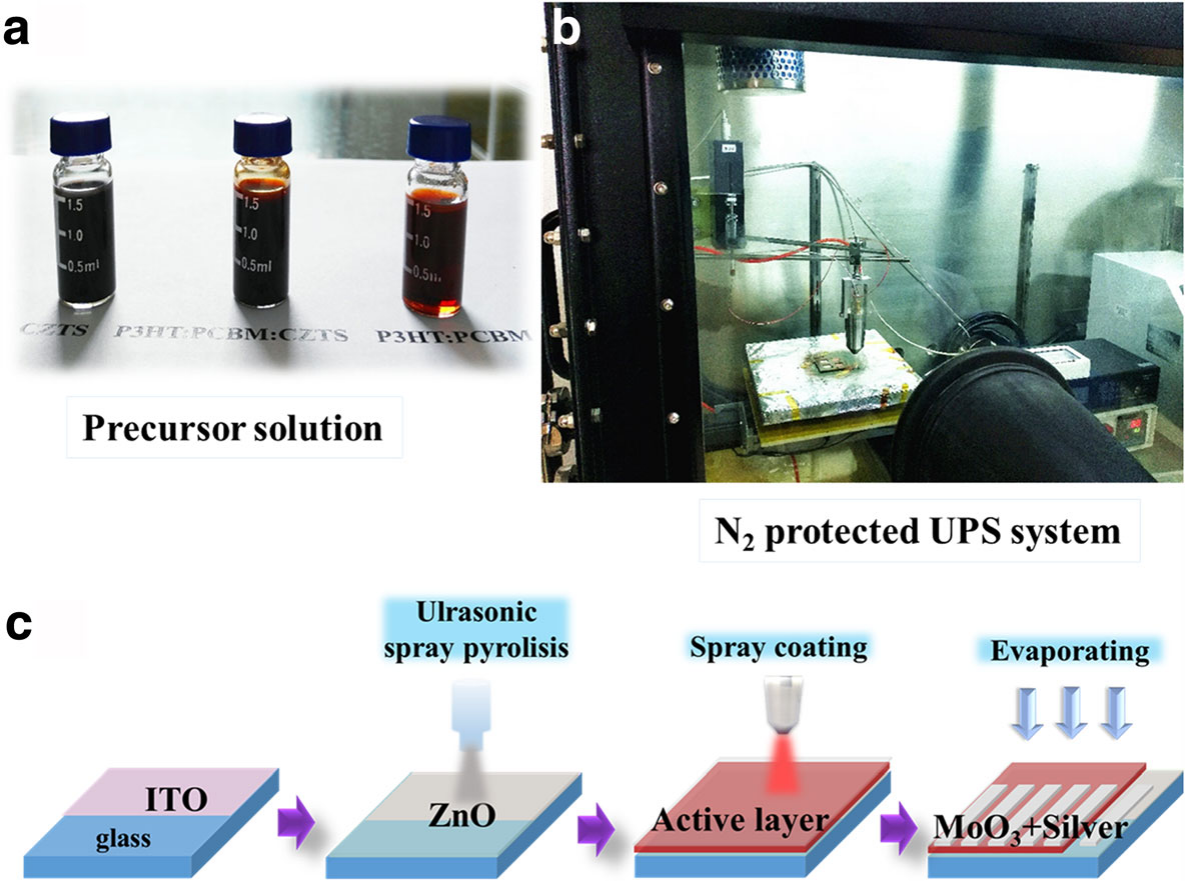
**a** CZTS NCs and P3HT:PCBM dispersed in o-DCB solution. **b** Glove-box protected ultrasonic spray system. **c** Fabrication process of photovoltaic devices

Glass/indium tin oxide (ITO) substrates were cleaned stepwise in an ultrasonic bath containing a detergent, acetone, deionized water, and ethanol (each step for 10 min) and then dried with nitrogen. The preparation steps of CZTS/polymer solar cells are shown in Fig. 1c. A 50-nm ZnO layer was grown on the ITO by using the previously reported ultrasonic spray pyrolysis method at 200 °C [19, 20]. Consecutively, the precursor solution was deposited by spray coating to form a ~350-nm-thick absorber layer by using an ultrasonic spray system (nozzle: Siansonic Z95S). The active polymer solution was prepared by dissolving 5 mg P3HT (Rieke Metals) and 5 mg PCBM (Nano-C) in 1 mL o-dichlorobenzene (ODCB). Subsequently, different concentrations of CZTS NPs were added to the solution. Before the active layer deposition, hybrid solutions were sonicated for at least 30 min. A MoO_*x*_ film (8 nm) and an Ag film (150 nm) were then successively thermally evaporated onto the hybrid blend at a pressure below 10^−4^ Pa. Four samples were prepared in a process, each sample having five cells with an active area of 0.06 cm^2^

### Characterization of CZTS Nanocrystals and Layers

The composition and crystal structure were characterized by X-ray diffraction (XRD, Bruker D8) in a conventional mode. The nanoscale information of CZTS NPs and blend absorbers were characterized by scanning electron microscopy (SEM, Zeiss Merlin) and high-resolution transmission electron microscopy with selected-area electron diffraction (SRED) (HRTEM; Zeiss Libra200). The NC composition was characterized by Raman spectroscopy (LabRam HR800 UV, Horiba Jobin-Yvon) with an excitation wavelength of 514 nm. Optical transmittance spectra in the 340–720 nm wavelength range were carried out on blend films by using a UV-Vis-IR spectrophotometer (Agilent Cary 5000). The film thickness was measured by a stylus profilometer (Alpha-Step D-100). Current density–voltage (*J*–*V*) characteristics of photovoltaic devices were recorded with a Keithley 2400 under a xenon lamp (7IS0503A, Beijing SOFN) with an illumination power of 100 mW/cm^2^. The external quantum efficiency (EQE) was measured using an integrated system (7-SCSpecIII, Beijing SOFN) and a lock-in amplifier with a current preamplifier under short-circuit conditions.

## Results and Discussion

### Crystal Structure and Composition of NPs Using Different Chemical Stabilizers

**Fig. 2:**
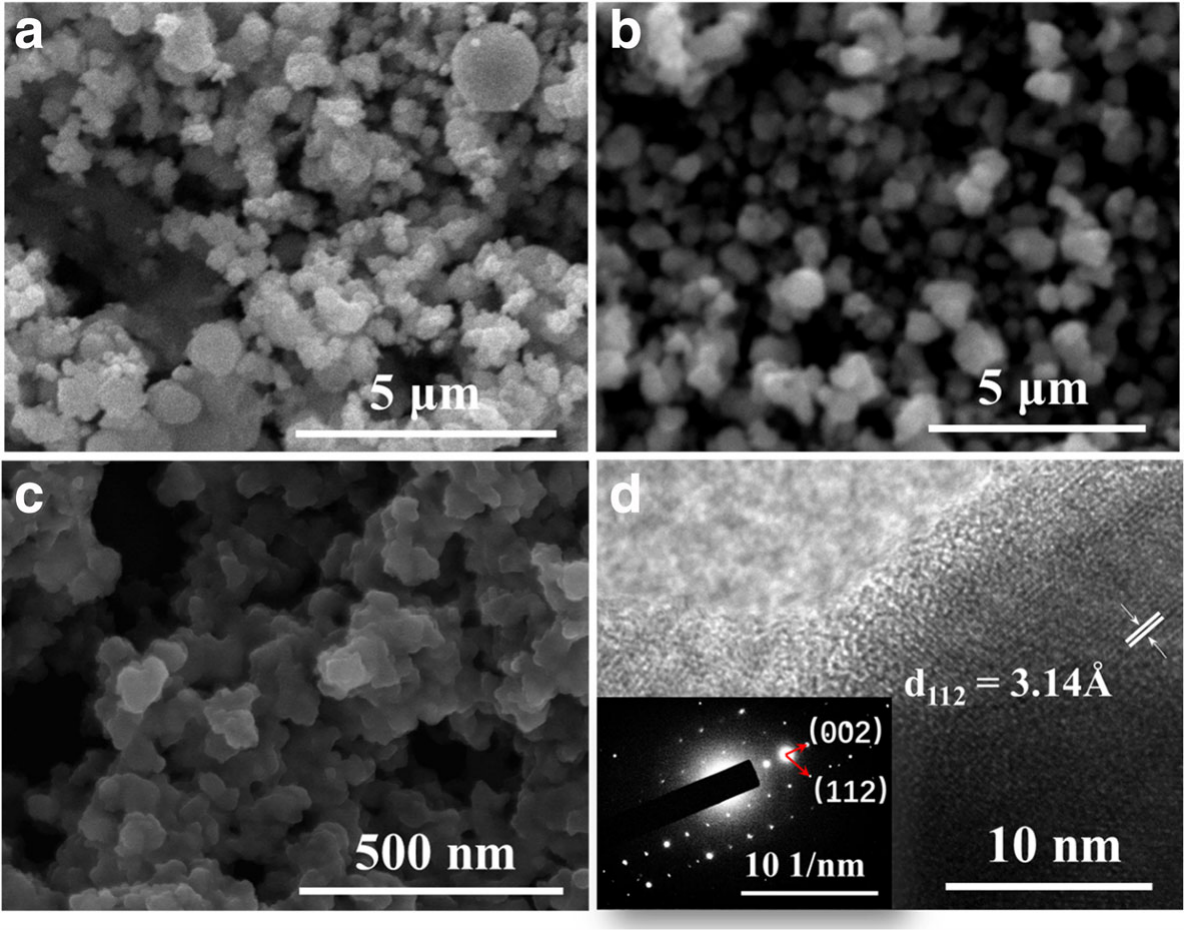
SEM image of as-synthesized nanoparticles using different chemical stabilizers: **a** 3 mL ethanolamine, **b** 1.5 mL ethanolamine + 1.5 mL oleylamine, **c** 1.5 mL ethanolamine + 1.5 mL diethanolamine, and **d** HRTEM image with SRED of the sample in **c**

For the conventional solvothermal method, the difficulty is to obtain single-phase monocrystalline NPs from a solution including Cu^+^, Zn^2+^, and Sn^4+^ cations. One function of the chemical stabilizer is to promote the cation dissolution in alcohols. An even more important role is to modulate the cation crystallization temperature. As we see in the SEM image Fig. 2a, by using the precursor with 3 mL pure ethanolamine chemical stabilizer, a multiphase final product showing different grain shapes was obtained. When the chemical stabilizer was replaced by 1.5 mL ethanolamine + 1.5 mL oleylamine, or 1.5 mL ethanolamine + 1.5 mL diethanolamine, final products with different grain sizes were obtained. Remarkably, when using a mixture of ethanolamine and diethanolamine, NPs displayed a consistent shape and a suitable size for being dispersed in the active photovoltaic layer. With other stabilizers, the NP size was even larger than the target thickness of the active layer.

**Fig. 3:**
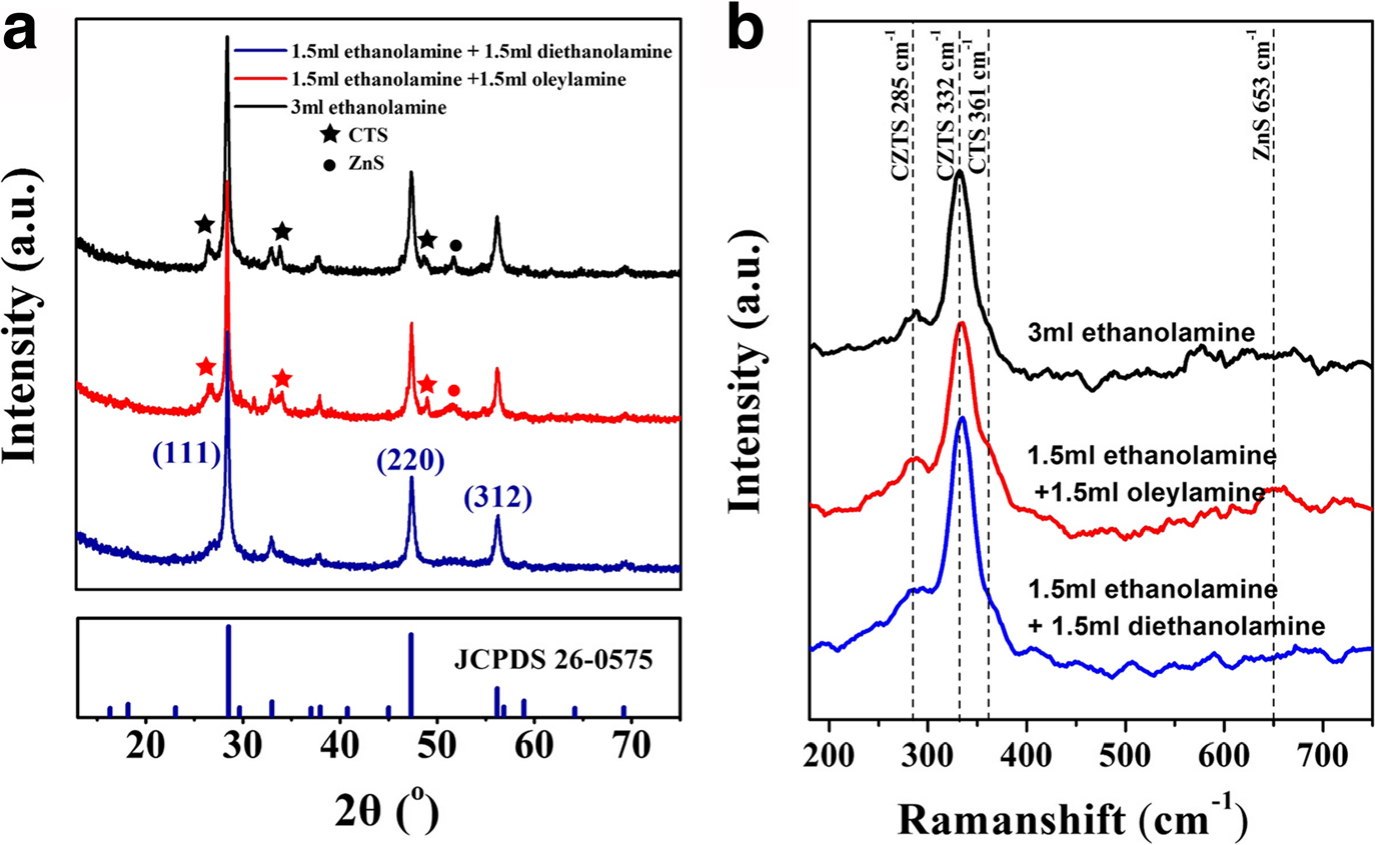
XRD patterns (**a**) and Raman spectra (**b**) of as-synthesized NPs using different chemical stabilizers

XRD patterns are shown in Fig 3a. Main diffraction peaks ascribed to the (112), (220), and (312) planes of CZTS are easily identified all the three patterns. Since lattice parameters of CZTS are close to those in CuSnS_3_ (CTS) and ZnS, it is difficult to distinguish among these compounds by looking at diffraction peaks. However, several CTS- and ZnS-related diffraction peaks can be obviously identified in the nanopowder synthesized by using ethanolamine or the ethanolamine and oleylamine mixture as a chemical stabilizer. Raman analysis provides for a more univocal composition assignment as shown in Fig. 3b. Two CZTS Raman peaks at 332 and 285 cm^−1^ are detected, in good agreement with previously reported results [21], confirming that each of the three powder samples is mainly composed of CZTS. It is worth noting that, in agreement with XRD results, CTS and ZnS Raman modes, respectively, at 361 cm^−1^ and 653 cm^−1^, were simultaneously found only in samples synthesized using ethanolamine or the ethanolamine/oleylamine mixture as a chemical stabilizer.

**Fig. 4:**
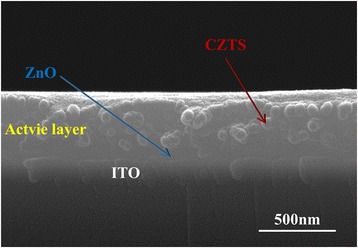
Cross-sectional SEM image of P3HT:PCBM:CZTS hybrid blend film deposited by ultrasonic spray

According to our experience, single-phase and well-dispersed CZTS NPs can be achieved using the binary chemical stabilizer system composed of ethanolamine and diethanolamine with a 1:1 volume ratio. As shown in Fig 3b, when the 1.5 mL ethanolamine and 1.5 mL diethanolamine chemical stabilizer was used, no other characteristic peaks from impurities were observed in the Raman spectrum. In Fig. 2a, the SEM image shows a consistent NP shape with an average grain size of about 50 nm. Figure 2d shows the HRTEM image and SRED pattern of CZTS NPs. The HRTEM image shows that NPs have clear lattice fringes with an interplanar spacing of 3.14 Å; this was ascribed to the (112) plane and well agrees with the XRD peak at 28.6^o^ (Fig. 2a), demonstrating the crystalline nature of CZTS NCs. SRED pattern is composed by periodically arranged diffraction spots consistent with the CZTS lattice, indicating the CZTS domains are monocrystalline. In addition, the composition and the average grain size are appropriate for the absorber layer fabrication. As-synthesized CZTS NPs were dispersed in P3HT:PCBM solution following the scheme in Fig. 1a. In the deposition process, an ultrasonic spray system was employed to provide for a higher curing rate for the hybrid solute at a very low N_2_ flow rate. Accordingly, polymer-wrapped particles do not diffuse during solvent evaporation. If a spin-coating method was used, NPs would tend to move to the wafer edge under the centrifugal force, resulting in an inhomogeneous final NP dispersion and surface morphology. As shown in Fig. 4b, the ultrasonic spray deposition resulted in well-dispersed CZTS NPs into the active blend layer. The cross-sectional SEM of the active layer shows a homogeneous NP dispersion and a uniform thickness. This resulted in devices which were not susceptible to short circuits and with a good yield of more than 95% (for CZTS ≤2 mg/mL).

### Photovoltaic Performances

**Fig. 5:**
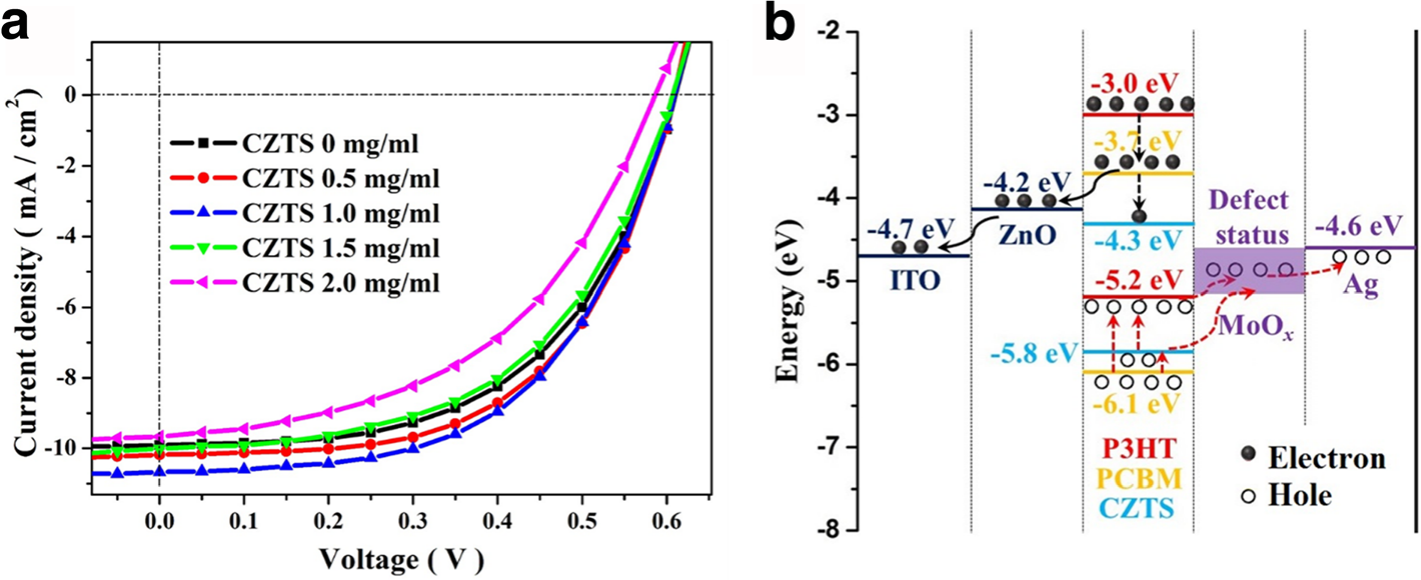
*J*–*V* characteristics under illumination (**a**) and energy levels of the P3HT:PCBM:CZTS hybrid solar cells (**b**)

Typical *J*–*V* characteristics of photovoltaic devices assembled with different monocrystalline CZTS NP concentrations under illumination are shown in Fig. 5. Device performances are summarized in Table 1. The standard device, without NPs, shows an open-circuit voltage (*V*_OC_) of 0.61 V, a short-circuit current density (*J*_SC_) of 9.90 mA/cm^2^, a fill factor (FF) of 54.61%, and a PCE of 3.30%. The *J*_SC_ was obviously improved even with the addition of a small amount CZTS NPs. For a CZTS NP concentration of 1.0 mg/mL, the *J*_SC_ reached a maximum value of 10.67 mA/cm^2^. The optimum device performance was obtained for a CZTS NP concentration between 0.5 and 1.0 mg/mL, with a best PCE of 3.65%, corresponding to a 10.6% enhancement with respect to the standard device. Interestingly, for a higher CZTS NP concentration of 1.5 mg/mL, the device performance showed a degradation of the PCE and a lower FF and shunt resistance (*R*_SH_). For a higher CZTS NP concentration of 2 mg/mL, the PCE was further reduced to 2.75% with a FF of 48.51%.

**Table 1:** Solar cell parameters obtained with different CZTS NP concentrations

CZTS (mg/mL)	*V*_OC_ (V)	*J*_SC_ (mA/cm^2^)	FF (%)	PCE (%)	*R*_S_ (Ω cm^2^)	*R*_SH_ (Ω cm^2^)
0	0.61	9.90	54.61	3.30	13	1565
0.5	0.61	10.38	56.71	3.58	11	1527
1.0	0.61	10.67	55.09	3.65	13	1473
1.5	0.61	10.21	52.86	3.27	14	789
2.0	0.59	9.67	48.51	2.75	17	629

In order to investigate the photovoltaic performance enhancement mechanism, the PSC energy level alignment has been drawn (Fig. 5b). Under light irradiation, both the active polymer and CZTS NPs do absorb photons and generate excitons [18]. Excitons are split into electrons and holes in the P3HT-PCBM and CZTS-PCBM heterojunction interface. Then, electrons are transferred to the PCBM lowest unoccupied molecular orbital (LUMO) level and holes to the P3HT highest unoccupied molecular orbital (HOMO) level of the CZTS. Thanks to the convenient energy level and the high carrier mobility, holes in CZTS can be easily and quickly transported to the MoO_*x*_ and be collected at the Ag electrode. However, the LOMO level of CZTS (−4.3 eV) is lower than that of ZnO (−4.2 eV), meaning that electrons in the CZTS are blocked from transporting the cathode, resulting in the accumulation of electron in the active layer [16, 22]. When a small amount of CZTS (~1 mg/mL) is added to the active layer, the advantage of carrier’s generation was greater than its deficiency in carrier transmission. Therefore, the device acquired a subsequent higher *J*_SC_ and quantum efficiency. On the other hand, when the active layer had more CZTS NPs (i.e., 2 mg/mL), the electron transport deficiency effect could not be any more ignored. Carrier recombination rate reaches to an unfavorable level; thus, the device PCE declined gradually with the obviously drop of *R*_SH_.

**Fig. 6:**
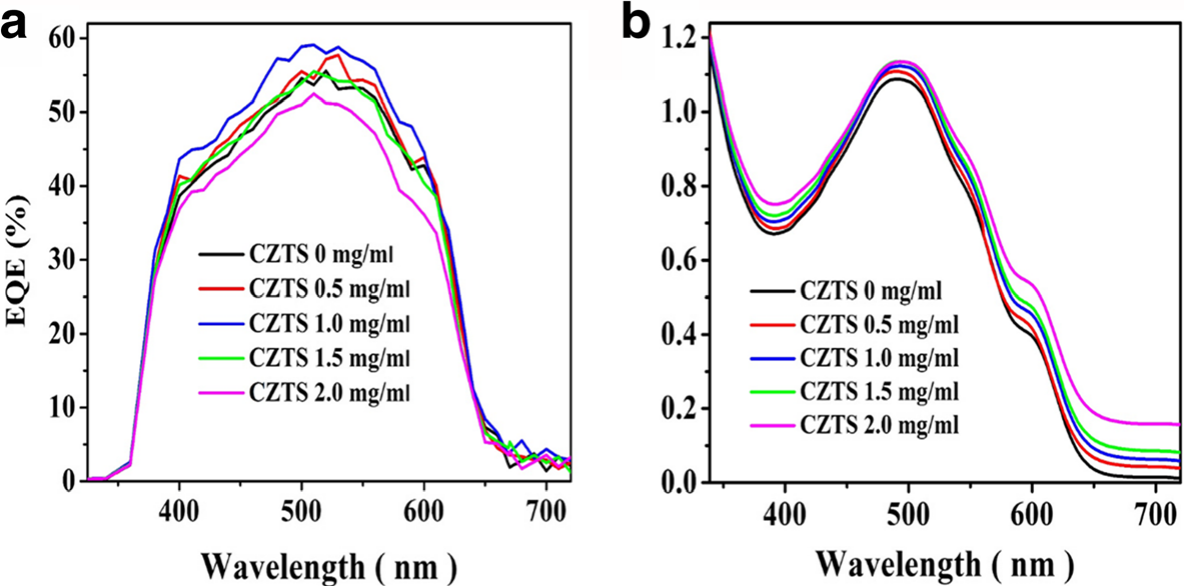
**a** EQE of solar cells and **b** UV-Vis-IR absorption spectrum of blend films containing different concentrations of CZTS NPs

To study the light-harvesting performance, the light absorption and the EQE were simultaneously tested. According to the EQE spectrum shown in Fig. 6a, devices with 0.5 to 1.0 mg/mL CZTS NPs result in a clear improvement in the EQE. The EQE increment is thought to rely mostly on the higher light absorbance. As shown in Fig. 6b, the blend film UV-Vis-IR absorbance shows a gradual increment with the CZTS NP content. The EQE increment was observed at almost all the entire tested wavelength range; this is ascribed to the higher light absorption in the broad (400–1000 nm) spectral range [21, 23], indicating CZTS NPs modify the light-harvesting capability of the P3HT:PCBM layer.

**Fig. 7:**
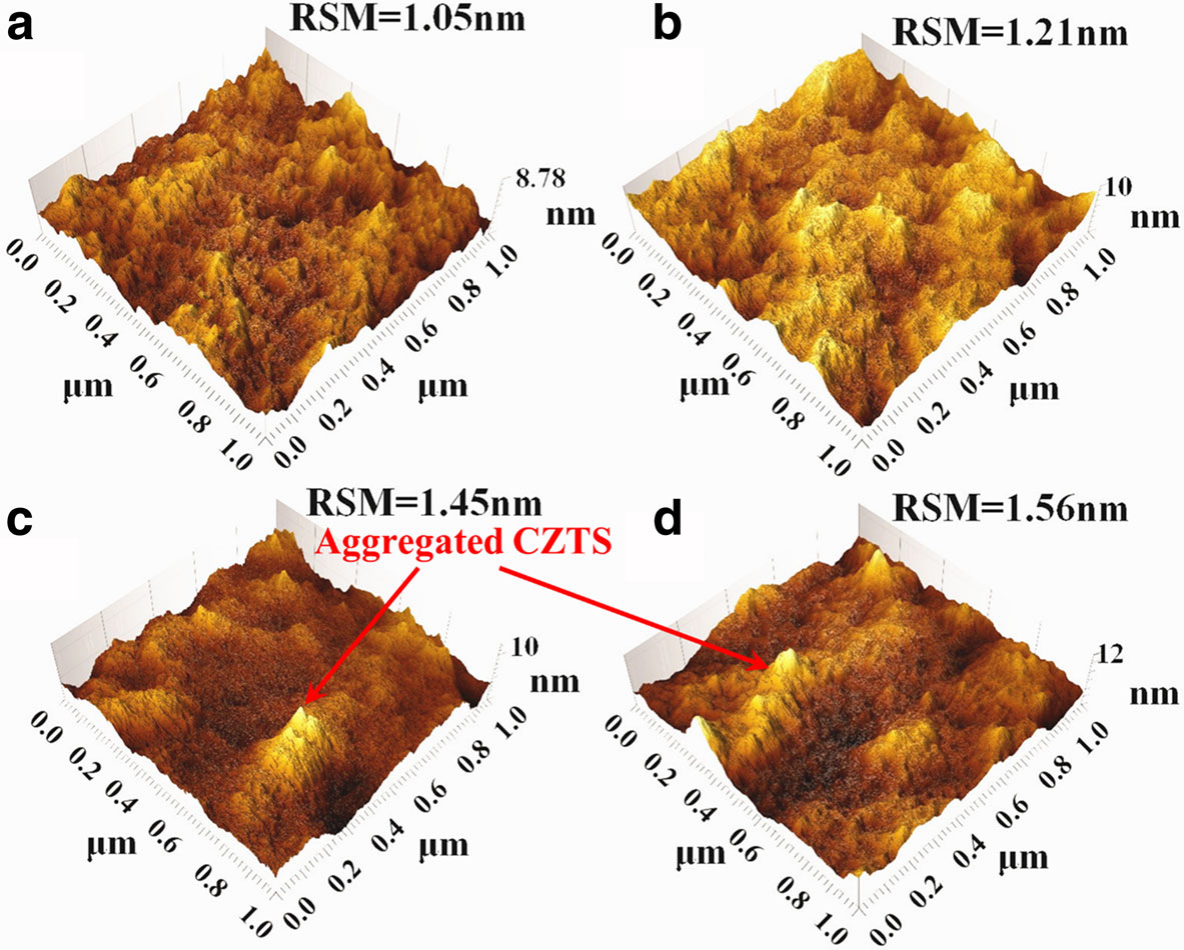
AFM image of blend films with different CZTS NP concentrations. **a** 0 mg/mL. **b** 0.5 mg/mL. **c** 1.5 mg/mL. **d** 2.0 mg/mL

It is well-known that the interface between active layer and metal electrode is crucial to the carrier’s collection [24]. AFM was used to observe how the morphology of blend active layer is affected by CZTS NP concentration. AFM images allow displaying the 3D topography and extracting the surface roughness (Fig. 7). The root mean square (RMS) roughness value is 1.05 nm in the bare P3HT:PCBM film, and it increases with the CZTS NP concentration when this is varied from 0.5 to 2.0 mg/mL. However, the RMS value increase is weak, as the roughest surface was only 1.56 nm, which is much lower what previously reported for P3HT:PCBM:FeS_2_ films [16]. When the NP concentration is 1.5 and 2.0 mg/mL, some aggregated particle ridges appear at the surface of the active layer. Although a low increase in the surface roughness can be beneficial to the light absorption [25, 26], due to the presence surface defects acting as recombination sites for the photo-generated charge, charges tend to be captured by these aggregated particle ridges rather than be transported to the metal electrode [27], resulting to a lower *R*_SH_ and a lower FF in the solar cell. We think that this is an additional cause leading to the degradation of devices with a large CZTS NP concentration.

## Conclusions

High-quality and well-dispersed CZTS nanoparticles have been successfully prepared by a solvothermal method using an appropriate chemical stabilizer containing ethanolamine and diethanolamine with the volume ratio of 1:1. As-synthesized CZTS NPs resulted to be well-dispersed in a hybrid polymer/fullerene aromatic solution as well as in spray-deposited hybrid active layers. By adding a small amount of CZTS to photovoltaic active layers, the hybrid device resulted in an enhanced light-harvesting ability and a 10.6% enhancement of the PCE with respect to the reference device without CZTS NCs. This approach has the advantage of being environmental friendly, cost-effective, and compatible with large-scale production.

## Abbreviations

AFM: Atomic force microscope
BHJ: Bulk heterojunction
CTS: CuSnS_3_
CZTS: Cu_2_ZnSnS_4_
EQE: External quantum efficiency
HOMO: Highest unoccupied molecular orbital
HRTEM: High-resolution transmission electron microscopy
ITO: Indium tin oxide
*J*_SC_: Current density
*J*–*V*: Current density–voltage
LUMO: Lowest unoccupied molecular orbital
NC: Nanocrystal
NP: Nanoparticle
o-DCB: o-Dichlorobenzene
P3HT: Poly 3-hexylthiophene-2,5-diyl
PCBM: [6,6]-Phenyl C61 butyric acid methyl ester
PCE: Power conversion efficiency
PPL: Para poly phenol
PSC: Polymer solar cells
RMS: Root mean square
*R*_SH_: Shunt resistance
SRED: Selected-area electron diffraction
*V*_OC_: Open-circuit voltage
XRD: X-ray diffraction
*η*: Photo conversion efficiency
